# Evaluation of anti-Müllerian hormone in pre-menopausal women stratified according to thyroid function, autoimmunity and age

**DOI:** 10.1186/s13044-022-00133-5

**Published:** 2022-08-15

**Authors:** Massimo Giusti, Miranda Mittica

**Affiliations:** 1 Endocrine Unit, Clinical Diagnostic Centre Priamar, via dei Partigiani 13R, 17100 Savona, Italy; 2 Department of Internal Medicine, University of Genova, Genoa, Italy; 3 Local Health Service 3 Genovese, Liguria Region, Genoa, Italy

**Keywords:** Thyroid function, Thyroid autoimmunity, Thyroid volume, AMH, Pre-menopausal women

## Abstract

**Background:**

AMH is a reliable index of ovarian reserve. It is not clear whether, or how much, thyroid function and/or thyroid autoimmunity can impair ovarian function and AMH secretion in the long term.

**Aim:**

This retrospective cross-sectional study compared AMH levels in pre-menopausal women with/without positive thyroid autoimmunity or hypofunction.

**Methods:**

From January 2019 to May 2022, AMH was evaluated in 250 pre-menopausal women not undergoing assisted fertility procedures who were referred to a secondary endocrine centre. Thyroid function and autoimmunity, sonographically measured thyroid volume, FSH and E2 in the early follicular phase, and PRL and progesterone in the luteal phase were also evaluated. Exclusion criteria were: age < 18 years, genetic hypogonadism, pregnancy and previous treatments that have potentially damaging effects on gonads.

**Results:**

We evaluated 171 women (mean age ± SD: 31.5 ± 9.0 years) off L-T4 treatment and 79 women on L-T4 treatment (39.7 ± 9.5 years; *P* < 0.001). AMH (median, IQR, CI) was 16.1 pmol/l (7.1 – 35.7 pmol/l, 21.4 – 29.9 pmol/l) and 7.6 pmol/l (1.4 – 17.8 pmol/l, 8.6 – 14.7 pmol/l; *P* < 0.001), respectively. When the women were stratified according to age (18-25, 26-30, 31-35, 36-40, 41-45, > 46 years) no significant difference emerged between those on/off L-T4 treatment in groups of the same age-range. In women on- or off-L-T4 treatment, AMH was negatively related with age on univariate and multivariate analyses (*P* < 0.0001). In both groups, AMH was negatively related to FSH (*P* < 0.0001). On multivariate analysis, AMH was positively related to the age of the mother on spontaneous menopause (*P* = 0.006) and negatively to thyroid volume (*P* = 0.02) in women on L-T4. AMH levels were significantly (*P* = 0.03) higher in TPOAb-negative than in TPOAb-positive women, but age was significantly (*P* = 0.001) lower in TPOAb-negative than in TPOAb-positive women.

**Conclusions:**

In our cohort of women, age proved to be a better predictor of AMH levels than any of the other factors linked to thyroid function and autoimmunity. Our data do not support the hypothesis that subclinical hypothyroidism and/or autoimmunity are associated with decreased ovarian reserve. However, a larger number of cases is needed in order to obtain conclusive data.

## Introduction

The anti-Müllerian hormone (AMH) is produced by granulosa cells of pre-antral and antral follicles in the ovaries, and AMH reflects the ovarian reserve [1, 2]. Therefore, the number of follicles recruited during the fertile age is believed to be directly related to the size of the primordial follicle pool [3]. AMH is considered to be the most accurate marker of the growing follicle pool and ovarian function [1, 2]. Genetic, hormonal, nutritional and environmental factors, surgical procedures, exposure to ionizing radiation and toxic drugs and other unknown factors impair ovarian function over time in the reproductive age [4]. Age is the main factor related to the secretion of AMH, which decreases by about 5-7 pmol/l every 3-5 years in the fertile period [2, 5].

Thyroid dysfunction is the most common endocrine disorder in women of reproductive age. While overt hypothyroidism and hyperthyroidism may cause menstrual abnormalities [6], it is debated whether subclinical thyroid diseases can cause ovarian dysfunction and infertility [7, 8]. Although current guidelines stress the importance of evaluating thyroid autoimmunity and function in the pre-gestational and gestational periods [9], adherence to interventional treatment in sub-clinical hypothyroidism in real life is still sub-optimal [8]. Closer surveillance of thyroid function is commonly adopted in women who are evaluated for infertility and trying to conceive through assisted reproductive techniques (ART). Murto et al. [10] identified thyroid-stimulating hormone (TSH) values < 2.5 mIU/l and AMH > 10 pmol/l as significant predictors of live births in women with unexplained infertility.

A literature search conducted at the end of 2021 identified several papers in which AMH and thyroid function and/or autoimmunity were evaluated. The studies involved had mainly been conducted in centres for human reproduction, and no agreement emerged on the role of the thyroid in AMH secretion or indirectly on the ovarian reserve [11–21]. In 2015, a study involving a large number of women undergoing screening for infertility, in which a cross-sectional retrospective analysis of AMH, free-thyroxine (f-T4), TSH and thyroperoxidase antibody (TPOAb) was conducted, concluded that thyroid autoimmunity and function were not associated with low ovarian reserve [12]. By contrast, a 2020 study involving a large number of infertile women over 35 years of age documented a diminished ovarian reserve in sub-clinical hypothyroidism [17]. Recently, in a case-control study conducted on a small sample of women of reproductive age in an endocrine setting, AMH was reported to be decreased in chronic autoimmune thyroiditis, independently of the type or titres of anti-thyroid antibodies [21].

In our region, AMH is currently assessed in only two public centres that implement ART. However, AMH evaluation is not generally requested by gynaecologists and endocrinologists in their assessment of the functioning of the pituitary-gonadal axis, except in pre-menopausal women with a history of radioiodine treatment for thyroid cancer [22]. The present study was conducted in a secondary Ligurian endocrinological setting. We retrospectively searched for AMH data on pre-menopausal women as a marker of ovarian function in several medical conditions besides ART. Our objective was to study thyroid determinants (thyroid function, treatments, autoimmunity, thyroid volume) of AMH serum values in an endocrinological setting, in order to verify the hypothesis of differences in AMH levels in women with or without thyroid dysfunctions.

## Material and methods

### Study design and subjects

**Fig. 1:**
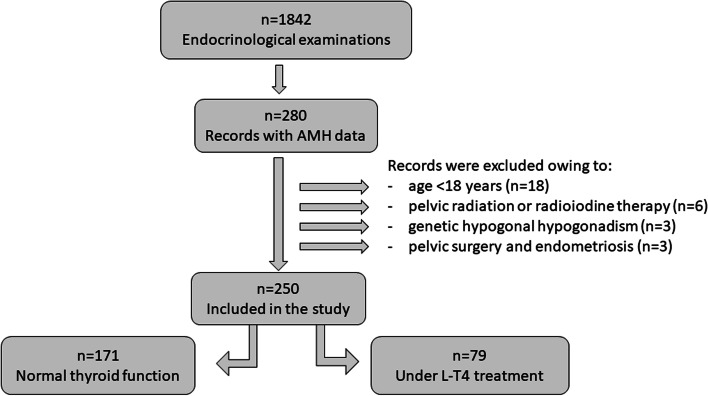
Flowchart of the study

This retrospective cross-sectional study was conducted at the Endocrine Unit of Priamar Clinical Diagnostic Centre, a private secondary-level outpatient centre located in the Savona district (Liguria, Italy). Endocrinological examination was mostly requested by general practitioners or other specialists, and sometimes directly by the patient. The examination was requested mainly for thyroid, metabolic and pituitary-gonadal health problems. Women who had undergone at least one AMH evaluation performed in association with functional (f-T4, TSH), autoimmune (TPOAb) and ultrasonography (US) thyroid evaluation were anonymously picked out from 1691 medical files collected in the period 2019-2022. AMH data were retrieved from 280 files of pre-menopausal women not undergoing ART procedures. Exclusion criteria were: age < 18 years, genetic hypogonadism, pregnancy, pelvic surgery, and previous treatments that have potentially damaging effects on gonads. Data on 250 women (Fig. 1) were included in the analysis; these women were off (*n* = 171) or on (*n* = 79) levo-thyroxine (L-T4) treatment for sub-clinical hypothyroidism. Some women were on L-T4 following surgical thyroidectomy (*n* = 16) or for goitre (*n* = 5). In women with spontaneous menstrual cycles (*n* = 186), blood samples were collected on days 2-4 of the menstrual cycle for AMH, follicle-stimulating hormone (FSH) and 17β-oestradiol (E2) assay, and on days 21-24 for progesterone and prolactin (PRL) assay. In women (*n* = 35) with menses induced by oral contraceptives (*n* = 28) or by medroxyprogesterone acetate (*n* = 7; 100 mg/orally), the ovarian reserve and PRL were evaluated at the time of thyroid evaluation. In women (*n* = 29) under evaluation for secondary amenorrhoea, AMH, E2, FSH and PRL were evaluated as part of diagnostic screening before treatments. AMH was evaluated along with full assessment of the pituitary-gonadal axis.

In accordance with general guidelines [23] in which 5-year age-groups were used in order to correlate data with age in women of reproductive age, we divided women into subgroups according to age (18-25, 26-30, 31-35, 36-40, 41-45, > 46 years). Owing to the retrospective nature of the study, some clinical data were missing (Table 1). AMH and thyroid function were the primary outcomes. The secondary outcomes included the other parameters linked to ovarian function and thyroid autoimmunity. Thyroid volume was considered a supplementary outcome.

**Table 1:** Some clinical and thyroidal data evaluated. The numbers of women in whom the given parameters were available are reported in brackets. Comparison was made between women with normal thyroid function and women on L-T4 therapy. Other drugs were: spironolactone (*n* = 2), dopamine-antagonist (*n* = 2), steroids (*n* = 2), rizatriptan (*n* = 1), and hydroxychloroquine (*n* = 1)

	All women (***n*** = 250)	Women with normal thyroid function (***n*** = 171)	Women on L-T4 therapy (***n*** = 79)	Significance ***P***
Age (year); mean ± SD	34.1 ± 9.9	31.5 ± 9.2	39.7 ± 9.5	< 0.0001
BMI (kg/m^2^); median, IQR, Cl	22.2; 20.2-25.6 21.5-22.9	21.7; 19.9-25.2 21.1-22.6	23.5; 21.2-26.6 21.9-22.4	0.03
Non-smokers (%)	71% (116)	72% (90)	54% (26)	0.01
Former/current smokers (%)	29% (47)	28% (25)	46% (22)	
TSH (mIU/l); median, IQR, Cl	2.00; 1.28-2.84 1.84-2.23	2.08; 1.35-2.83 1.87-2.30	1.84; 1.05-2.85 1.48-2.17	0.11
f-T4 (pmol/l); median, IQR, Cl	13.4; 11.5-15.7 13.0-14.0	12.9; 11.1-14.7 12.4-13.3	15.2; 12.8-17.6 14.0-16.2	< 0.0001
L-T4 (μg/day); median, IQR, Cl	–	–	60.0; 50.0-90.7 57.1-75.0	
TPOAb positive (%)	43% (166)	20% (96)	66% (70)	< 0.0001
TV (ml); median, IQR, Cl	7.9; 6.4-10.6 (215) 7.4-8.4	7.9; 6.6-10.3 (151) 7.6-8.5	8.0; 5.4-12.1 (64) 6.2-9.5	
Other ongoing drugs (number) none	124	91	33	
vitamin D/other vitamins	27	14	13	
hormonal contraceptives	28	21	7	
psychotropic drugs	23	13	10	
cabergoline	17	13	4	
metformin	8	6	2	
anti-secretive drugs	7	2	5	
H1- inhibitors drugs	5	4	1	
statins	3	2	1	
other drugs	8	7	1	

### Methods

Body mass index (BMI) was calculated on the basis of the weight (kg) and height (m) reported in medical files, according to the following formula: kg/m^2^. Smoking habits were investigated and women were classified as non-smokers, former smokers and smokers. Thyroid volume (TV) was calculated by means of US, as previously reported [24], by using the depth, width and length of each lobe reported in medical files. TV was obtained by combining the volumes of both lobes. All US examinations were performed by the same experienced endocrinologist (MG) with a UF-850 XTD Fukuda Denshi machine (Tokyo, Japan) equipped with linear probes working at 7.5 MHz. Intra-observer variability was 11.5%. In our district, normal TV in women is 8.0 ml (IQR 6.7 – 9.8 ml; range 3.2-19.8 ml) [24].

### Assays

All samples were drawn in the morning in the fasting condition. As previously reported [25], serum AMH was measured by means of a fully-automated two-site immunoassay based on the combination of ruthenium electrochemiluminescence and streptavidin-biotin technology (ECLIA; Elecsys® AMH, Roche Diagnostics, Milan, Italy). The functional sensitivity is 0.21 pmol/l (to convert to ng/ml, divide by 7.13). Within-run imprecision, repeatability and intermediate precision, all expressed as coefficients of variation (CV, %), are 0.5-1.8%, 1.7-2.6%, 2.1-2.9%, respectively. The expected values in pre-menopausal women on days 2-3 of the menstrual cycle range from 9.3 to 105.5 pmol/l. FSH, E2, PRL and progesterone were measured by means of enzyme-enhanced chemiluminescent immunoassays [25]. The expected values of FSH and E2 in the early follicular phase were less than 14.4 IU/l and 308.7 pmol/l, respectively. The expected values of PRL and progesterone in the mid-luteal phase were less than 25 μg/l and more than 12.7 nmol/l, respectively. Free-T4 (f-T4; normal range 12.0-22.0 pmol/l) and TSH (normal range 0.3-4.2 mIU/l) were evaluated by electrochemiluminescence immunoassay, optimized on the Cobas platform (Roche Diagnostics, Milan, Italy). The TSH functional sensitivity is 0.01 mIU/l, with intra- and inter-assay imprecision of 3 and 7%, respectively [25]. Several commercial methods were used for TPOAb evaluation during the study period, and judgements of negative TPOAb values were assigned according to the normal range reported by the manufacturers.

### Statistical analysis

Menstrual cycles with an interval of 28 ± 2 days and 3-5 days of bleeding were deemed regular and ovulatory when progesterone levels in the mid-luteal phase were > 12.7 nmol/l. GraphPad 9.0 software (GraphPad, San Diego, CA, USA) was used for statistical analysis. The absence of normality in AMH levels was tested by means of the Kolmogorov-Smirnov test. To compare continuous data, the Mann-Whitney test was used. Percentages were compared by means of Chi-square or Fisher’s exact test. Correlations were evaluated by means of univariate (Spearman test) and multivariate (Least squares) correlations. Data are reported as mean ± standard deviation (SD), median, IQR, and confidence limit (Cl). Significance was set at *P* < 0.05. The range of significance between 0.1 and < 0.0001 is reported.

### Ethical approval

Owing to the retrospective nature of the study, no formal approval from the Liguria Ethics Committee was required. All patients had provided written informed consent before their examinations and had agreed to the use of their clinical data for scientific research. Data collection and subsequent analysis were performed in compliance with the Helsinki Declaration.

## Results

Table 1 shows some clinical data on the subjects studied. The median age of all the subjects who underwent AMH analysis was 34.1 years, ranging between 18 and 55 years. Women on L-T4 therapy were significantly older (age range 18-55 years) than those with normal thyroid function (18-53 years; *P* < 0.0001) because the need for L-T4 therapy increased with age. The median (IQR) age was 42 years (30-47 years) in women on L-T4 and 32 years (24-38 years) in women off L-T4. Regarding BMI, there was a significant (*P* = 0.03) difference between the two groups of women, though BMI was slightly higher in older women. The percentage of current/former smokers was significantly higher (*P* = 0.01) among women on L-T4 therapy. TSH and TV were similar in both groups of women, while f-T4 and the percentage of women with positive TPOAb were significantly (*P* < 0.0001) higher in women on L-T4 therapy. There was no significant difference between the two groups in the percentage of other ongoing drugs (Table 1). Table 2 shows AMH levels and some hormonal data on the pituitary-gonadal axis. AMH (*P* < 0.0001) and FSH (*P* = 0.007) levels were significantly lower in women on L-T4 treatment. PRL levels did not differ significantly between the groups. Samples collected from normal cycling women in the theoretical luteal phase were available in 62% of cases. The percentages of ovulatory cycles (progesterone > 12.7 nmol/l) were similar in women with normal thyroid function (68%) and in those on L-T4 therapy (65%). Progesterone levels were similar in women on L-T4 treatment and in those with normal thyroid function (Table 2).

**Table 2:** AMH, FSH, E2, progesterone and PRL levels in all women and in women subdivided according to normal thyroid function or on L-T4 treatment. Data are reported as median, IQR and Cl. The numbers of women in whom the given parameters were available are reported in brackets. Comparison was made between women with normal thyroid function and women on L-T4 therapy

	All women (***n*** = 250)	Women with normal thyroid function (***n*** = 171)	Women on L-T4 therapy (***n*** = 79)	Significance ***P***
AMH (pmol/l)	12.0; 4.0 – 29.0 9.9-16.1	16.1; 7.1 – 35.7 12.2-24.5	7.5; 1.4 – 22.4 4.3-9.9	< 0.0001
FSH (IU/l)	7.4; 5.5 - 10.8 (185) 6.8 – 7.9	6.9; 5.2 - 9.3 (124) 6.1 – 7.6	8.6; 6.5 -13.0 (61) 7.8 – 10.7	0.007
E2 (pmol/l)	150.5; 113.8 - 209.3 (185) 139.5 – 168.9	146.8; 113.8 – 209.3 (126) 135.8 – 168.9	157.9; 113.8 – 220.3 (59) 139.5 – 183.6	
Progesterone (nmol/l)	23.9; 7.0 -37.8 (143) 20.0 – 25.8	22.9; 4.1 – 35.0 (91) 17.5 – 25.8	25.1; 14.9 – 41.3 (52) 19.4 – 30.5	
PRL (μg/l)	14.0; 8.5 -21.2 (186) 12.3 – 16.2	14.1; 8.0 – 20.9 (130) 11.7 – 16.7	13.4; 9.1 -25.0 (56) 11.4 – 16.8	

When the women (*n* = 165) were stratified according to negative or positive TPOAb results, AMH levels were significantly (*P* = 0.03) higher in TPOAb-negative (*n* = 105) (13.3 pmol/l; 5.3 – 24.7 pmol/l, Cl 9.4 – 18.8 pmol/l) than in TPOAb-positive (*n* = 60) (8.7 pmol/; 2.5 – 20.9 pmol/l) women, but age was significantly (*P* = 0.001) lower in TPOAb-negative (33.8 ± 9.2 years; median, IQR: 34 years, 26.0-41.5 years; range 18-51 years) than in TPOAb-positive (38.6 ± 9.2 years; median 40.0 years, 30.8-46.8 years; range 18-52 years) women.

**Fig. 2:**
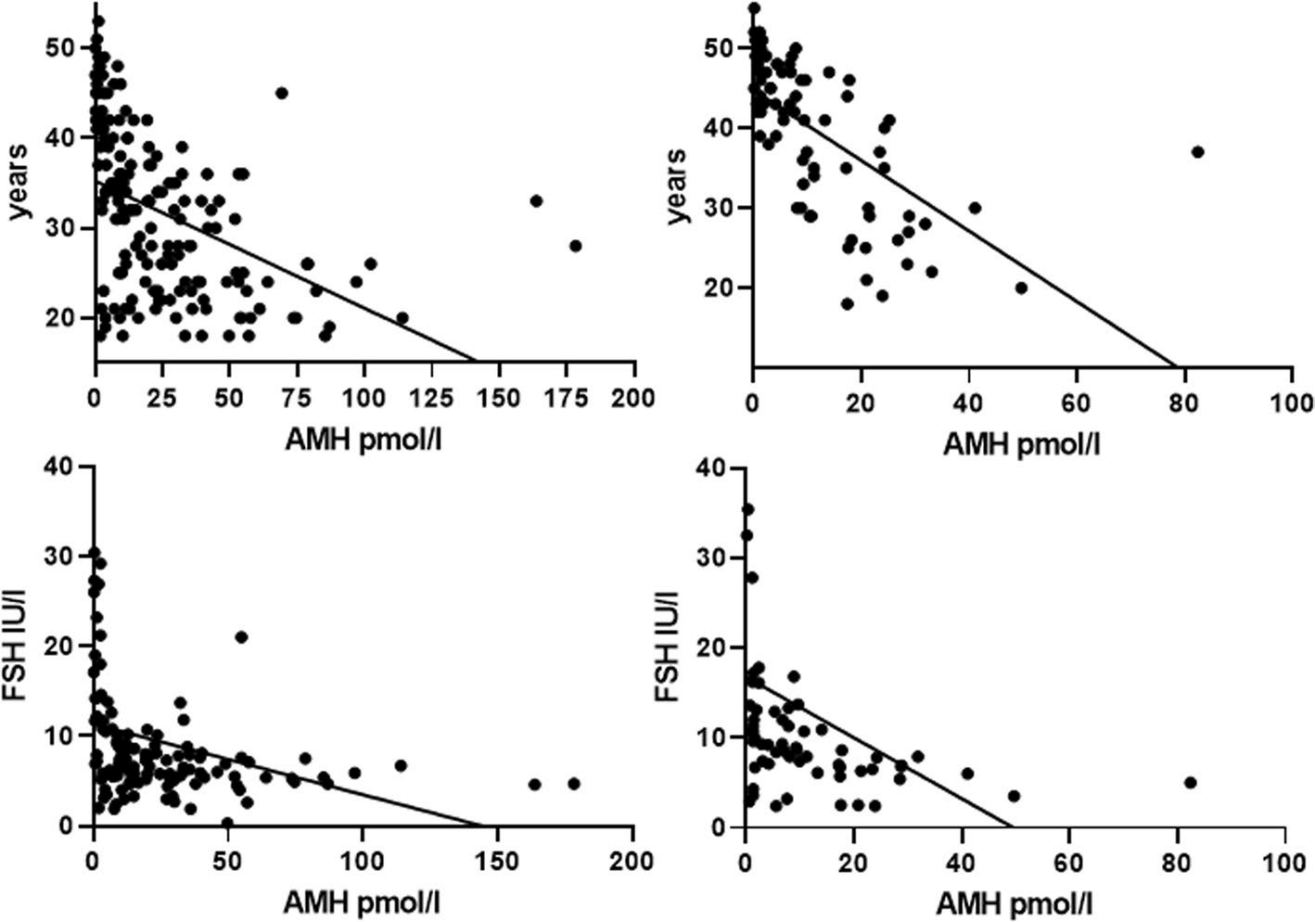
Correlation between AMH and age (top) or FSH levels (bottom) in the subgroups of women with normal thyroid function (left side) and in those on L-T4 (right side)

The univariate correlation of AMH with age, BMI, mother’s age on spontaneous menopause, hormonal parameters, TV and L-T4 dosage are reported in Table 3. AMH and FSH were negatively and significantly (*P* < 0.0001) related with age, both when evaluated in all women (Table 2) and when evaluated in women on/off L-T4 treatment (Table 2, Fig. 2). In all women, AMH was also significantly related with BMI (*P* = 0.05) (Table 3). On multivariate analysis, the dependent variable AMH remained significantly related with age both in all women (t 4.07, *P* = 0.0002) and in women off (t 2.70, *P* = 0.01) or on L-T4 treatment (t 5.23, *P* = 0.0001). Only in hypothyroid women on L-T4 was AMH related positively with the age of the mother on spontaneous menopause (t 3.18, *P* = 0.006) and negatively with TV (t 2.53, *P* = 0.02) on multivariate analysis.

**Table 3:** Univariate correlation of AMH levels with the other clinical and biochemical parameters evaluated in all women and in the subgroups of women with normal thyroid function and those on L-T4 therapy

	All women			Women with normal thyroid function			Women on L-T4 therapy		
	Pair (n)	Sr	Significance ***P***	Pair (n)	Sr	Significance ***P***	Pair (n)	Sr	Significance ***P***
Age	250	−0.69	< 0.0001	171	−0.59	< 0.0001	79	−0.76	< 0.0001
BMI	244	−0.12	0.05	167	−0.07	–	77	−0.07	–
TSH	230	0.03	–	154	−0.05	–	76	0.09	–
f-T4	198	−0.11	–	128	−0.02	–	70	−0.01	–
L-T4	–	–	–	–	–	–	79	−0.17	0.1
Menopausal age in mother	151	−0.04	–	98	−0.12	–	53	0.09	–
FSH	185	−0.53	< 0.0001	123	−0.45	< 0.0001	62	−0.58	< 0.0001
E2	185	−0.11	0.1	125	−0.12	–	60	−0.10	–
Progesterone	141	−0.10	–						
PRL	186	−0.10	–	129	−0.18	0.06	57	0.24	0.08
TV	215	0.00	–	152	−0.08	–	63	0.20	0.1

**Fig. 3:**
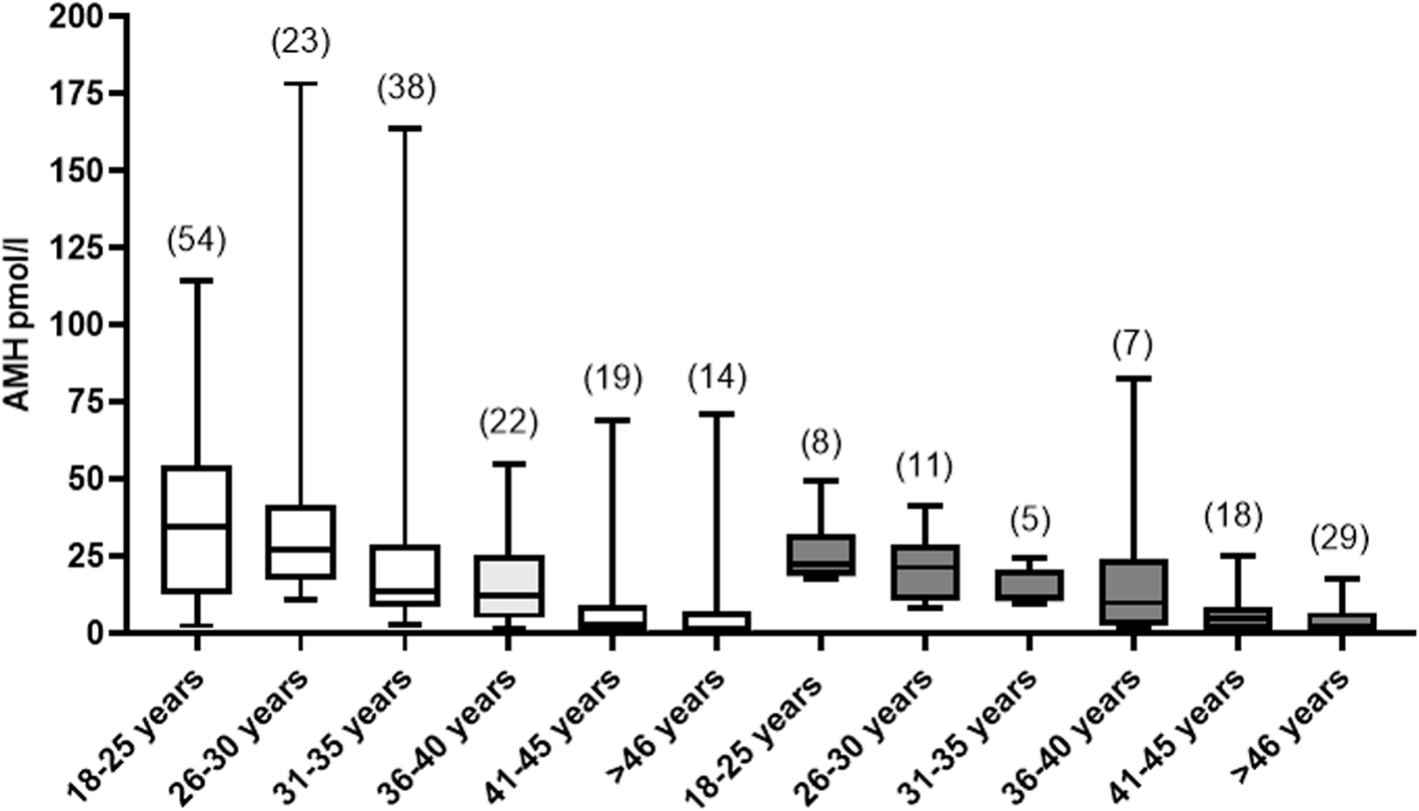
Median (IQR and range) of AMH levels observed in the subgroups of women with normal thyroid function (empty bars) and in those on L-T4 (filled bars), stratified according to age. The number at the top of each bar indicates the number of women in each age-group

Figure 3 reports AMH levels observed in women arbitrarily divided into subgroups according to age. In each subgroup, AMH levels did not differ significantly between women with normal thyroid function and hypothyroid women on L-T4 therapy (Fig. 3).

## Discussion

This retrospective single-centre study documents that AMH is sometimes assayed in our district as part of pituitary-gonadal evaluation. This is probably due to the authors’ previous interest in AMH [22] and does not reflect the habitual endocrinological management of the pituitary-gonadal axis in our district. This is in contrast with the reported indication for ovarian testing in certain situations (e.g. polycystic ovarian syndrome, peri-menopause, infertility, prior to ovarian surgery in reproductive-age women) besides ART, before ovarian stimulation [23].

It is well known that age is the most important factor related to the secretion of AMH, which decreases by about 5-7 pmol/l every 3-5 years in the fertile years [2, 5]. Tal and Davies [23] reported the lower limits of age-appropriate serum AMH values, stratified in 5-year intervals: 3.0 ng/ml at 25 years, 2.5 ng/ml at 30 years, 1.5 ng/ml at 35 years, 1 ng/ml at 40 years and 0.5 ml at 45 years. These levels are quite similar to those observed at the 25th percentile in comparable quintiles in our population (25-30 years: 16.9 pmol/l; 31-35 years: 8.9 pmol/l; 36-40 years: 5.0 pmol/l, and 41-45 years: 1.26 pmol/l). We also observed a very strong inverse correlation between age and both AMH and FSH in our women of reproductive age, independently from thyroid function and autoimmunity. The number of subjects and the size of the age-range can influence this observation. For instance, in 314 Iranian women with a mean age of 36.7 years (±SD, 6.1 years), Kabodmehai et al. [26] reported that older age correlated very significantly (*P* < 0.0001) with low AMH, a finding that was similar to ours. Moreover, in 67 consecutive infertile Japanese women with a mean age of 35.0 years (±SD 3.5 years; range not given) Kuroda et al. [11] reported that age correlated negatively with AMH levels, while in 27 normal fertile women of a similar median age (34.0 years) and with an age-range of 30-39 years, it did not. Finally, in women aged 20-40 years, Kucukler et al. [27] reported a significant inverse correlation between AMH and age both in those with normal thyroid function and in those with newly diagnosed sub-clinical or overt thyroid dysfunction. By contrast, on evaluating AMH levels in women aged 18-35 years, Adamska et al. [20] did not find any age-related changes in AMH in either 46 normal or 39 euthyroid TPOAb-positive women in this age-range.

It is well known that the incidence of autoimmune thyroiditis increases with age. Therefore, it is not surprising that our TPOAb-positive women had lower AMH levels than their TPOAb-negative counterparts, as their age was greater. Samsami et al. [19] stratified women into two age-groups: < 35 years old and > 35 years old, and found that AMH levels were lower only in TPOAb-positive women in the latter group. That autoimmune damage to the ovaries might take longer to become detectable is suggested by a longitudinal study which showed that women with a low ovarian reserve had higher baseline levels of TPOAb, and that these levels increased over 12-year follow-up [16].

In our pre-menopausal women, who were arbitrarily divided into subgroups aged from 18 years to > 46 years, AMH levels did not differ between women with normal thyroid function and hypothyroid women on L-T4 therapy. Similar results emerged from the study by Polyzoz et al. [12]. These authors evaluated AMH levels by means of a non-sensitive immunoassay (functional sensitivity 2.5 pmol/l) in women stratified by age (range not reported; mean age 32 years) and categorized ovarian reserve as low (<10th percentile), normal or high (>90th percentile) according to age-specific AMH levels; they found that TPOAb did not differ significantly among groups [12].

In our study, the absence of differences in age-specific AMH levels between women off/on L-T4 treatment could be due to the normalization of thyroid function in this latter group. Moreover, there was no correlation between AMH levels and TSH and f-T4 levels, nor, in L-T4 treated women, between L-T4 dosage and AMH. Then again, the impact of L-T4 treatment on AMH levels is debated. In the study by Öztürk Ünsal et al. [21], in which 39% of women with chronic autoimmune thyroiditis were on L-T4 treatment, AMH concentrations were similar in patients on/off L-T4. By contrast, Kuroda et al. [14] found that AMH levels in 35 women with Hashimoto’s disease had improved after 3 months of L-T4 treatment.

Control of body weight could be another factor in preserving ovarian function. However, data on the interrelationship between BMI and AMH levels are still debated. In our study, a weak negative correlation between AMH and BMI was observed in all women; this remained significant on multivariate analysis only in hypothyroid women on L-T4, whose BMI was higher. A significant negative correlation between BMI or waist circumference and AMH was reported in a Turkish study involving a small number of women with normal thyroid function and sub-clinical or overt hypothyroidism when they were cumulatively evaluated [26]. Adamska et al. [20] also reported a negative correlation between serum AMH and the percentage of body fat mass, as estimated by bioimpedance analysis, in a group of 39 women with Hashimoto’s thyroiditis, but not in 46 control women. By contrast, no correlation between AMH and BMI was observed in two older studies [11, 18]. Recently, AMH was evaluated in women with polycystic ovarian syndrome, and was found to be significantly and negatively correlated with BMI and waist-to-height ratio [28]. The mechanisms underlying this inverse correlation between AMH and BMI are still unclear, though the impact of insulin resistance on follicular development in PCOS has been speculated [29]. Interestingly, it has been suggested that the inverse relationship between serum levels of AMH and BMI could be the result of hormone dilution due to higher blood volume in women with elevated BMI [20].

For several years, we have sought to define normal TV in our Ligurian population [24]. Moreover, when taking our patients’ history, we routinely ask women if they know the age at which their mothers’ menopause occurred. Consequently, these data are almost always reported in our medical files. Interestingly, in the present study, AMH was significantly related to TV (negatively) and the age of spontaneous menopause in the mother (positively) on multivariate analysis. The former finding could be linked to the time-related reduction in TV on L-T4 treatment [30], and the latter to genetic factors [23]. Only Adamska et al. [20] reported TV in their study on AMH; they found no difference in TV between women with Hashimoto’s thyroiditis and control women of same median age of 26 years, the median value being 10 ml, which is only slightly higher than that found in our women (8 ml). This difference in TV could be explained by the difference in age-range, goitre control by means of L-T4 treatment, and regional differences in iodine intake.

Our study has several limitations. The first lies in the relatively small number of women involved and the retrospective design, which made it impossible to assess any temporal relationship between AMH and thyroid function/autoimmunity. In addition, as our women were recruited in a single centre, a selection bias cannot be excluded. Endocrinological diagnoses in our women were heterogeneous (e.g. possible high AMH levels in PCOS patients) and factors other than thyroid function and autoimmunity may have influenced AMH levels. Finally, as our study did not include a group of women with untreated sub-clinical/overt hypothyroid, we cannot exclude the possibility that untreated hypothyroidism (i.e. elevated TSH levels) may be involved in the decline of AMH in women of reproductive age.

The strength of this study is that it evaluated AMH levels and thyroid parameters in a local endocrinological setting in several clinical endocrine conditions, and not only in women undergoing ART procedures.

In conclusion, in our cohort of women, age proved to be a better predictor of AMH levels than any of the other factors linked to thyroid function and autoimmunity. Our data do not support the hypothesis that sub-clinical hypothyroidism and/or autoimmunity are associated with decreased ovarian reserve. The role of BMI and thyroid volume should be better defined in a larger number of cases, in order to obtain conclusive data. Moreover, further research is needed in order to investigate thyroidal mechanisms that regulate AMH secretion and the ovarian reserve, even though two systematic reviews have recently been published [31, 32]. Finally, thorough endocrinological-metabolic evaluation should be carried out in order to facilitate the achievement of fertility when reproduction is desired [33].

## Data Availability

The datasets used and/or analysed during the current study are available from the corresponding author on reasonable request.

## Abbreviations

ART: Assisted reproductive techniques
AMH: Anti-Müllerian hormone
BMI: Body mass index
E2: 17β-oestradiol
FSH: Follicle-stimulating hormone
f-T4: Free-thyroxine
L-T4: Levo-thyroxine
PRL: Prolactin
TV: Thyroid volume
TSH: Thyroid-stimulating hormone
TPOAb: Thyroperoxidase antibody
US: Ultrasonography
